# Videolaparoscopic prostatectomy in porcine model for training residents

**DOI:** 10.1590/S1677-5538.IBJU.2021.0139

**Published:** 2021-06-20

**Authors:** Caio Vinicius Suartz, Rubens Pedrenho, Anderson Bruno Pellanda, Hiury Silva Andrade, Victor Srougi, Marco Antonio Arap, Anuar Ibrahim Mitre, Miguel Srougi, William Carlos Nahas, Ricardo Jordão Duarte

**Affiliations:** 1 Hospital das Clínicas da Faculdade de Medicina da Universidade de São Paulo Divisão de Urologia Unidade de Videolaparoscopia São PauloSP Brasil Unidade de Videolaparoscopia, Divisão de Urologia, Hospital das Clínicas da Faculdade de Medicina da Universidade de São Paulo – FMUSP, São Paulo, SP, Brasil.

## Abstract

**Introduction:**

Surgical training models prepare the resident for a more ethical surgical practice as well as providing a less steep learning curve. In urology, there are well-known models of pyeloplasty simulation, urethro-vesical anastomosis and nephrectomy, which have helped in the training of urology residents (1–3). Learning laparoscopic prostatectomy is a difficult surgery and requires advanced surgical skill from the surgeon (4), requires operate without a direct view of the surgical field in a two-dimensional space and with longer instruments (5).

Laparoscopic prostatectomy step by step makes the surgeon's learning curve less difficult, lead to less intraoperative complications, such as blood loss, while also enabling shorter operative time and less positive surgical margins (6).

The objective of surgical models is to simulate surgical procedures in a reliable way thus preparing the surgeon for his daily practice, surgical simulations in animal models have been described to compensate for inadequate clinical exposure (7).

The canine model of prostate cancer has many similarities with humans. Despite trying to develop a model that is as credible as possible, there are ethical issues in several countries, such as Brazil, that do not allow the use of live dogs for scientific experimentation and there is a difficulty in not standardizing the animals used (8, 9).

The swine surgical training model is widely known, accepted and used as a valuable tool in the teaching of new surgeons (10).

The porcine video laparoscopic prostatectomy model allows the urologist in training to exercise the skills required in a real surgical situation, practicing them in a single session (10). We will present an experimental model in pigs for training urology residents in laparoscopic radical prostatectomy with current techniques (11–13).

The limitations found are that the prostate has no limits as well defined as in humans, the urethra is long and coiled, the fat surrounding the pelvic organs is scarce and there is no postoperative follow-up for evaluating functionality after the procedure, as well as the effectiveness of the surgery with surgical margins. However, it is similar in surgical model presented, it is reproducible and can provide a realistic simulation environment to the beginner surgeon.

**Material and Methods:**

In this paper, according to the institutional protocol approved by the institutional ethics and research committee FMUSP n° 964/2017 and protocol was in accordance with current international regulations for the use of animals in Research: Reporting In Vivo Experiments (ARRIVE) guide. Ten male pigs weighing 20 to 22kg were used. The animals were anesthetized with a combination of Telazol (5mg/kg), Xylazine (1.5mg/kg), Cetamine (22mg/kg) and Atropine (0.04mg/kg) for orotracheal intubation followed by Isoflurane (2%). Animals were euthanized at the end of the procedure with a lethal dose of KCl (2mEq/kg). The trocar insertion points were marked using the epigastric vessels and umbilical region as reference points. Initially, urethral catheterization was performed using a hydrophilic Nitinol guidewire, followed by a perineal incision to dissect the tortuous urethra of the porcine model. A malleable urethral catheter 8Fr was inserted into their bladder. The animal was placed in the Trendelenburg position inserted and 12mm trocars were inserted in its umbilical region, utilizing 10mm in the surgeon's dominant hand, 5mm in his non-dominant hand of the surgeon, and 5mm in the first assistant's trocar.

The surgeon replicates the steps performed in a laparoscopic radical prostatectomy in humans, including the bladder catheterization, dissection of the anterior bladder plane, the vesicular and prostatic dissection, the suture of the dorsal venous plexus, a prostatectomy, an urethral vesical anast omosis, as well as the waterproof test, even including the performing of surgical steps using current concepts of anterior urethral suspension as the reconstruction of the posterior plane of the rhabdosphincter.

**Results:**

All steps of surgery could be reproduced in all ten porcine cases. No significant bleeding was observed and the surgical time was gradually reduced fifty percent from case one to last cases.

**Conclusions:**

The porcine model allowed the surgeon to replicate all the steps usually performed in a laparoscopic radical prostatectomy. The junior surgeons are better prepared to such difficult surgery. However, further studies will be necessary to prove the impact of the animal model presented in urological clinical practice.
